# Dr. Collie Again

**Published:** 1891-10-03

**Authors:** 


### DR. COLLIE AGAIN.

Sir,—An axiom which has been consistently advocated in your columns is the old "*audi alteram partem*"; permit me, therefore, to say that my daughter has but just been discharged from the Homerton Fever Hospital, after being detained there for about eleven weeks (suffering from scarlet fever, with a complication of rheumatic fever), and she has nothing but praise to give to Dr. Collie, Nurse Frazer, and the whole of the staff for the careful treatment and attention they have given to her. As I know nothing whatever of the officials, probably this testimony may discount a good deal of the "scandals" which, in such large institutions, are sure to arise to a greater or lesser extent from petty personal jealousies and spite.—I beg to remain, Yours faithfully,

Sussex.

